# Prevalence and allergy-related risk factors of overactive bladder in children in Northeast China

**DOI:** 10.3389/fpubh.2024.1415833

**Published:** 2024-10-04

**Authors:** Yue Zheng, Lu Yin, Xiuli Wang, Chengguang Zhao, Yue Du

**Affiliations:** Department of Pediatrics, Shengjing Hospital of China Medical University, Shenyang, China

**Keywords:** allergy, children, prevalence, overactive bladder, risk factors

## Abstract

**Objective:**

The objective of this study was to investigate the prevalence of overactive bladder (OAB) and its risk factors related to allergies among children in Northeastern China.

**Methods:**

A community survey on OAB was conducted in Northeast China from 1 April 2022 to 30 April 2022. The survey targeted children aged 5–14 years and utilized questionnaires. A total of 1,394 children were enrolled, and their parents were requested to fill out a questionnaire to provide basic information about the children. This encompassed details regarding the presence or absence of urgent urination unrelated to urinary tract infection, urinary tract infection, allergic rhinitis, asthma or cough variant asthma, atopic dermatitis, anaphylactic conjunctivitis, urticaria, constipation, and attention deficit hyperactivity disorder (ADHD). The prevalence of OAB was calculated. The chi-squared test was used to analyze OAB-related factors, which were subsequently included in the logistic regression model for multi-factor analysis.

**Results:**

The overall OAB prevalence was 10.7% (120 of 1,121), including 47 mild cases (39.2%), 71 moderate cases (59.1%), and 2 severe cases (1.7%). OAB prevalence decreased with age (*p* < 0.05). The risk factors associated with OAB were allergic asthma (OR = 1.87, 95%CI: 1.12–3.13), atopic dermatitis (OR = 2.45, 95%CI: 1.61–3.73), anaphylactic conjunctivitis (OR = 1.61, 95%CI: 1.07–2.42), and urticaria (OR = 1.93, 95%CI: 1.40–2.66).

**Conclusion:**

OAB prevalence among children in Northeastern China was found to be 10.7%, with its risk factors being allergic asthma, anaphylactic conjunctivitis, urticaria, and atopic dermatitis. The identification of allergy-related risk factors may provide new ideas for the prevention and treatment of OAB.

## Introduction

Overactive bladder (OAB) is a common lower urinary tract syndrome in children. The International Children’s Continence Society (ICCS) defines OAB as a syndrome characterized by urgent urination, which is often accompanied by frequent urination and nocturia, with or without urgent urinary incontinence, except in cases of urinary tract infections or other clear pathological changes (1). OAB prevalence in Chinese adults is approximately 6–11.3%, among which the prevalence in women is higher than that in men, increasing gradually with age and reaching more than 10% in subjects over 40 years of age (2–4). Of particular interest, OAB prevalence in children has been high in recent years, with its reported prevalence varying significantly among different ages and regions, ranging from 5 to 23% (5–7). Patients with OAB symptoms tend to stay away from social activities and interpersonal relationships, which affects their physical and psychological health and increases the economic and social burden on patients and their families. At present, the pathogenesis of OAB is mainly attributed to detrusor instability, bladder sensitivity, urethral and pelvic floor muscle dysfunction, mental and behavioral abnormalities, hormone metabolism disorders, etc. Previous evidence has also shown that dietary preferences are related to the incidence of OAB; the higher the protein intake, the lower the risk of developing OAB (8).

The pathogenesis of OAB in children is more complex than it is in adults. A previous study has provided some evidence that OAB is related to constipation, and these conditions have been collectively referred to as bladder and bowel dysfunction (BBD) (9). Allergic factors have been identified as significant contributors to the development of OAB in children, based on both clinical experience and previous research (10). The possible mechanism involves the infiltration of mast cells into the bladder wall. These mast cells not only directly mediate allergic or inflammatory responses, but also release mast cell tryptase (MCT). MCT then regulates protease-activated receptor 2 (PAR2), which enhances the sensitivity of the bladder’s afferent nerves, including bladder C-fiber sensitivity. This ultimately leads to bladder overactivity (11).

Therefore, we conducted a cross-sectional study with participants aged 5–14 years in Northeastern China to investigate OAB prevalence in children aged 5–14 years and to assess risk factors for allergy-associated OAB.

## Methods

### Research objects

This was a cross-sectional and analytical study. The enrolled study participants were from Northeast China, with ages ranging from 5 to 14 years. The inclusion criteria for the study were as follows: healthy children aged 5–14 years from Northeast China. The exclusion criteria for the study were as follows: (1) children with organic diseases such as urinary tract stones, tumors, diabetes, or diabetes insipidus; (2) children with a history of long-term medication use for chronic diseases. Their parents or legal guardians were interviewed from 1 April 2022 to 31 April 2022 for the purposes outlined below.

### Questionnaire

To collect data, a self-administered questionnaire was designed by the Department of Pediatric Nephrology and Rheumatology, Shengjing Hospital of China Medical University. It was based on The Overactive Bladder Symptom Score (OABSS) defined by the International Children’s Continence Society (ICCS) (12). The questionnaire was divided into three parts as follows: (1) Basic information, including age, sex, and dietary preferences (vegetarian, non-vegetarian, and balanced diet). (2) Evaluation of the absence or presence of urgent urination unrelated to a urinary tract infection. For individuals with urgent urination, the data collected were analyzed using the OABSS (Table 1): the number of daytime urinations per day, the number of nocturnal urinations, the frequency of sudden urgency to urinate with difficulty controlling urination, and the frequency of sudden urgency resulting in urinary incontinence. OAB was diagnosed when the urinary urgency score was ≥2 and the overall OABSS score was ≥3. A total OABSS score ≤5 was considered mild OAB; a total score of 6–11 was considered moderate OAB; and a total OABSS score ≥12 was considered severe OAB. (3) History of urinary tract infection, allergic rhinitis, asthma or cough variant asthma, atopic dermatitis, anaphylactic conjunctivitis, urticaria, constipation, and hyperkinetic syndrome (all diseases were diagnosed by specialists from upper second-class hospitals).

**Table 1:** Overactive bladder symptom score (OABSS) (11).

Question	Frequency	Score
Q1. How many times do you typically urinate from waking in the morning to going to sleep at night?		
	7 or less	0
	8–14	1
	15 or more	2
Q2. How many times do you typically wake up to urinate at night?		
	None	0
	1	1
	2	2
	3 or more	3
Q3. How often do you have a sudden desire to urinate that is difficult to defer?		
	None	0
	Less than once a week	1
	Once a week or more	2
	About once a day	3
	2–4 times a day	4
	5 times a day or more	5
Q4. How often do you leak urine because you cannot defer the sudden desire to urinate?		
	None	0
	Less than once a week	1
	Once a week or more	2
	About once a day	3
	2–4 times a day	4
	5 times a day or more	5

OAB, overactive bladder.

OAB, sum score ≥ 3 and score of Q2 ≥ 2; Mild-OAB, sum score ≤ 5; Moderate-OAB, 6 ≤ sum score ≤ 11; Severe-OAB, sum score ≥ 12.

### Data collection

**Figure 1:**
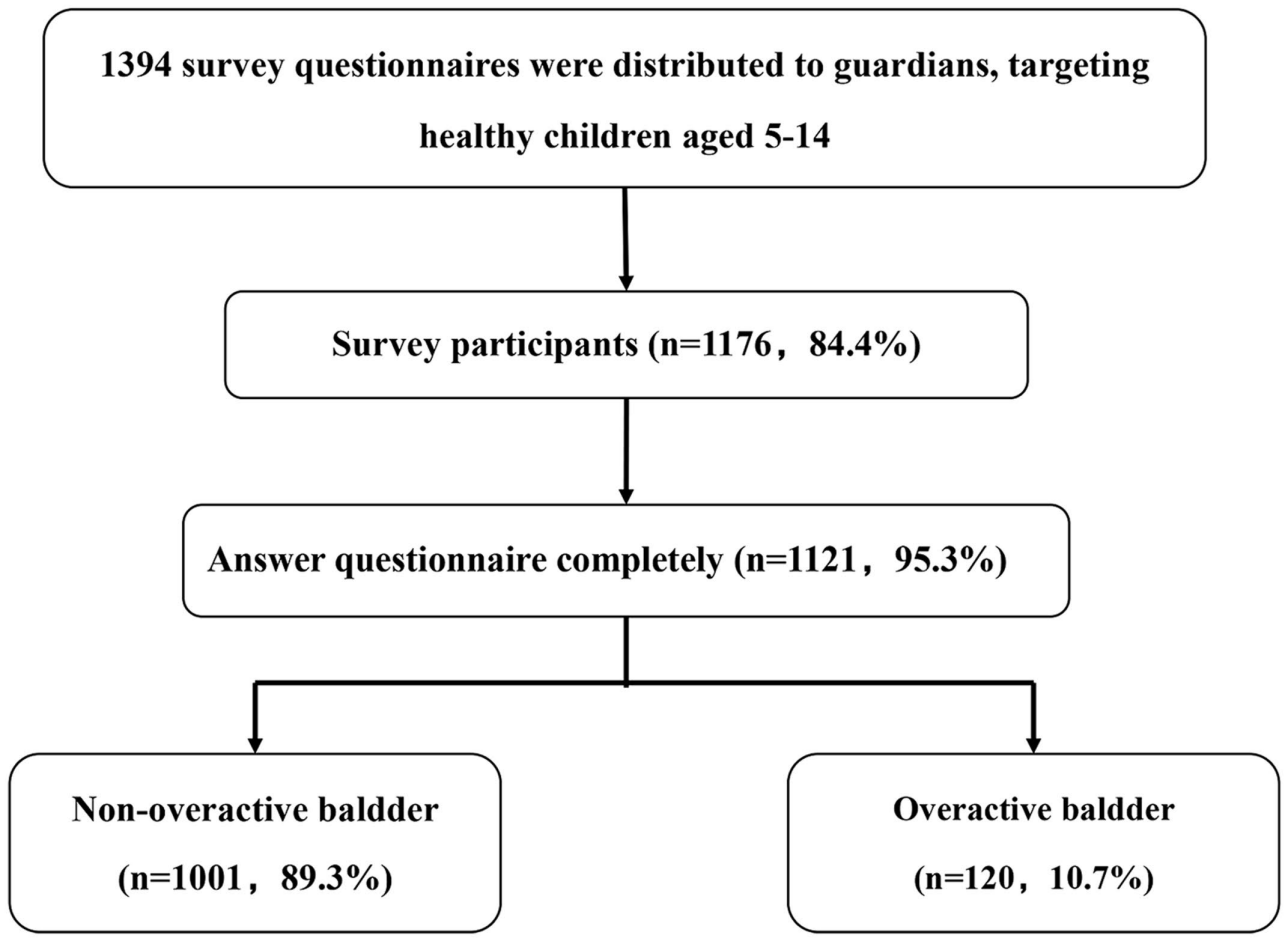
Flow diagram of the study. A total of 1,394 questionnaires were distributed, and 1,176 responses were received, yielding a response rate of 84.4%. A few participants with incomplete questionnaires or logical errors were excluded (*n* = 55). Ultimately, 1,121 participants were included in the final analysis.

A questionnaire weblink was generated on an online survey platform called “Wenjuanxing.” The survey was distributed to participants and their parents or legal guardians via email and WeChat, a popular instant messaging and social media application in China. Before assigning the survey, two investigators were trained as lead researchers. These trained researchers explained the details of the study to the participants and their parents or legal guardians. Then, they sent the questionnaire’s web page and the invitation to the participants and their parents or legal guardians. If the participants and their parents or legal guardians had any questions about the questionnaire, they could contact the investigators via email, phone, or WeChat. Each participant and their parents or legal guardian had the right to decide whether to participate in the study or to withdraw from the study at any time. Before the participants and their parents or legal guardians filled out the questionnaire, they were shown a page with the objectives of the study and were asked to give their informed consent. If the participants and their parents or legal guardians provided informed consent, they could proceed to complete the questionnaire. A primary researcher and two investigators examined each questionnaire for logical errors and missing items. The response rate was 84.4% (of 1,394 individuals, 1,176 responded). A few participants with incomplete questionnaires and logical errors were excluded (*n* = 55). A total of 1,121 participants were included in the final analysis (Figure 1).

### Data collation

We collected the baseline characteristics of the participants. OAB prevalence and the proportion of patients with mild, moderate, and severe OAB were counted. We calculated the incidence of OAB for different ages and sexes and performed risk factor analysis (RFA) by comparing data from the OAB and the non-OAB groups.

### Analytical methods

We used SPSS 25.0 software for statistical analysis. A *p*-value <0.05 was considered statistically significant. Measurement data are expressed as mean ± standard deviation, and count data are expressed as percent (%). The t-test was used for the comparison of measurement data between groups, and a chi-squared test (X2) was used for the comparison of count data between the groups. Univariate analysis was performed to examine the potential factors that may affect OAB, and binary logistic regression analysis was carried out on factors that indicated statistical significance in the univariate analysis.

### Medical ethics

This study was approved by the Ethics Committee of Shengjing Hospital, China Medical University (Ethics No. 2021PS581K). Written consent forms were signed by the parents or legal guardians of all participants.

## Results

Of the 1,121 participants, 628 were boys (56.02%) and 493 were girls (43.98%). The age distribution followed a normal distribution, with a mean age of 7.81 ± 2.46 years. The incidence of OAB was 10.7% (*n* = 120). According to the scoring criteria, there were 47 mild cases (39.2%), 71 moderate cases (59.1%), and 2 severe cases (1.7%). There were 62 boys (51.67%) and 58 girls (48.33%). The distribution of OAB by age group is shown in Table 2. The prevalence was higher in the 5–8 years age group (Table 2).

**Table 2:** The prevalence and baseline characteristics of OAB.

Characteristics	
**OAB characteristics**	120/1,121 (10.7%)
Mild	47/120 (39.2%)
Moderate	71/120 (59.1%)
Severe	2/120 (1.7%)
**Child characteristics**	
Age, mean ± standard deviation	7.81 ± 2.46
Sex of child, *n*/*N* (%)	
Boy	62/628 (9.9%)
Girl	58/493 (11.8%)
Age of child, *n*/*N* (%)	
5 years old	35/246 (14.2%)
6 years old	24/197 (12.2%)
7 years old	23/156 (14.7%)
8 years old	14/121 (11.6%)
9 years old	7/124 (5.6%)
10 years old	7/98 (7.1%)
11 years old	6/64 (9.4%)
12 years old	1/59 (1.7%)
13 years old	2/29 (6.9%)
14 years old	1/27 (3.7%)

OAB, overactive bladder.

This table shows that the overall prevalence of OAB in the study sample was 10.7%, with mild, moderate, and severe OAB accounting for 39.2%, 59.1%, and 1.7%, respectively. The average age of the children was 7.81 years, and the gender distribution indicated that the prevalence of OAB was 9.9% in boys and 11.8% in girls. The age distribution revealed a higher prevalence of OAB among children aged 5–8 years.

The distribution of OAB among individuals with other characteristics is shown in Table 3. In total, 117 participants had asthma (including cough variant asthma), and OAB prevalence with a history of asthma was 26 of 117 (22.2%). There were 230 cases with a history of atopic dermatitis, and OAB prevalence with a history of atopic dermatitis was 50 of 230 (21.7%). There were 361 cases with a history of anaphylactic conjunctivitis, and OAB prevalence with a history of anaphylactic conjunctivitis was 56 of 361 (15.5%). There were 146 cases with a history of urticaria, and OAB prevalence with a history of urticaria was 35 of 146 (24.0). There were 211 cases with a history of constipation, and OAB prevalence with a history of constipation was 31 of 211 (14.7%). There were 123 cases with a history of hyperactivity disorder, of which 23 cases (18.7%) met the criteria for OAB. OAB prevalence was not associated with risk factors such as sex, dietary preferences, and history of urinary tract infection. No abnormalities were observed in routine urinalysis.

**Table 3:** Results of the univariate analysis.

Characteristics	Total sample *N* = 1,121	OAB		
		OAB *N* = 120	Non OAB *N* = 1,001	*p* value
**Age**, mean ± standard deviation	7.81 ± 2.463	7.06 ± 2.055	7.89 ± 2.494	<0.001
**Sex, *N* (%)**				0.309
Boy	628 (56%)	62 (9.9%)	566 (90.1%)	
Girl	493 (44%)	58 (11.8%)	435 (88.2%)	
**Regular dietary preferences, *N* (%)**				0.528
Vegetarian diet	52 (4.6%)	8 (15.4%)	44 (84.6%)	
Meat diet	185 (16.5%)	20 (10.8%)	165 (89.2%)	
Balanced diet	884 (78.9%)	92 (10.4%)	792 (89.6%)	
**Past history of urinary tract infection, *N* (%)**				0.113
Yes	74 (6.6%)	12 (16.2%)	62 (83.8%)	
No	1,047 (93.4%)	108 (10.3%)	939 (89.7%)	
**Allergic rhinitis, *N* (%)**				0.193
Yes	317 (28.3%)	40 (12.6%)	277 (87.4%)	
No	804 (71.7%)	80 (10.0%)	724 (90.0%)	
**Asthma or cough variant asthma, *N* (%)**				<0.001
Yes	117 (10.4%)	26 (22.2%)	91 (77.8%)	
No	1,004 (89.6%)	94 (9.4%)	910 (90.6%)	
**Atopic dermatitis, *N* (%)**				<0.001
Yes	230 (20.5%)	50 (21.7%)	180 (78.3%)	
No	891 (79.5%)	70 (7.9%)	821 (92.1%)	
**Anaphylactic conjunctivitis, *N* (%)**				<0.001
Yes	361 (32.2%)	56 (15.5%)	305 (84.5%)	
No	760 (67.8%)	64 (8.4%)	696 (91.6%)	
**Urticaria, *N* (%)**				<0.001
No	975 (87%)	85 (8.7%)	890 (91.3%)	
Mild	102 (9.1%)	25 (24.5%)	77 (75.5%)	
Severe	44 (3.9%)	10 (22.7%)	34 (77.3%)	
**Constipation, *N* (%)**				0.038
Yes	211 (18.8%)	31 (14.7%)	180 (85.3%)	
No	910 (81.2%)	89 (9.8%)	821 (90.2%)	
**Hyperactivity disorder, *N* (%)**				0.002
Yes	123 (11%)	23 (18.7%)	100 (81.3%)	
No	998 (89%)	97 (9.7%)	901 (90.3%)	

OAB, overactive bladder.

This table presents the association between various characteristics and OAB through univariate analysis. The results show that gender, dietary preferences, history of urinary tract infections, and history of allergic rhinitis were not significantly associated with OAB (*p* > 0.05). However, allergic asthma, atopic dermatitis, allergic conjunctivitis, urticaria, constipation, and ADHD were significantly positively associated with OAB (*p* < 0.05).

**Figure 2:**
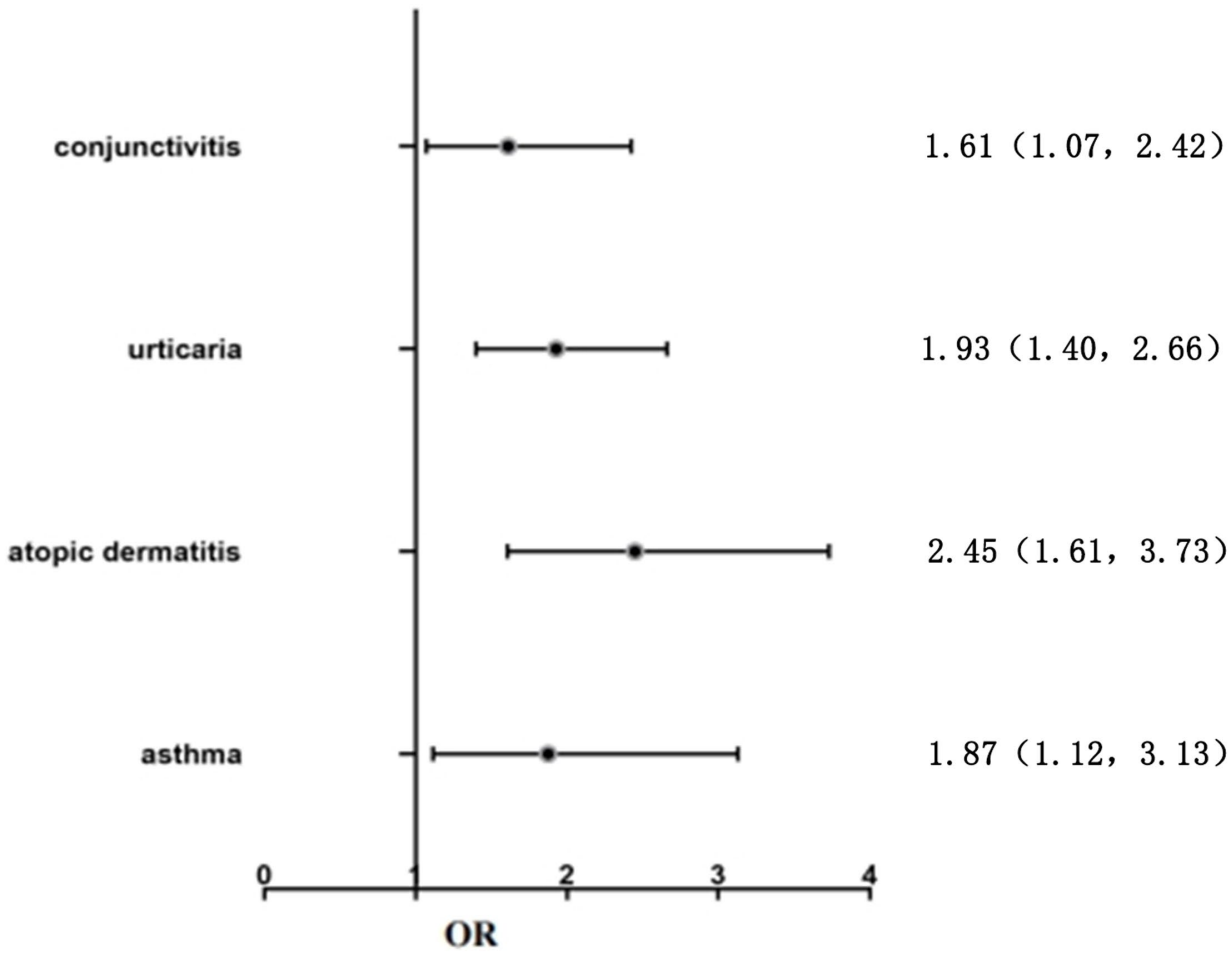
Logistic regression analysis of the risk factors in children with OAB.

Further investigation was conducted to examine the relationship between allergy-related factors and the occurrence of OAB. This analysis was based on the data presented in Table 4 and Figure 2. Asthma, atopic rhinitis, allergic rhinitis, urticaria, and other related conditions were included to construct a multi-factor logistic regression equation. The results indicated that children with a history of asthma had a higher likelihood of developing OAB compared to those without asthma. The difference was statistically significant (OR = 1.87, 95%CI: 1.12–3.13, *p* = 0.018). Atopic dermatitis in children caused an increased risk of OAB, and the difference was statistically significant (OR = 2.45, 95%CI: 1.61–3.73, *p* < 0.001). Anaphylactic conjunctivitis and urticaria both had a significant effect on OAB in children, and the differences were all statistically significant (OR = 1.61, 95%CI: 1.07–2.42, *p* = 0.022; OR = 1.93, 95%CI: 1.40–2.66, *p* < 0.05).

**Table 4:** Multivariate logistic regression results.

Factors	*B* value	*p* value	OR	95%CI
Allergic asthma	0.624	0.018	1.87	1.12, 3.13
Atopic dermatitis	0.895	<0.001	2.45	1.61, 3.73
Urticaria	0.657	<0.05	1.93	1.40, 2.66
Allergic conjunctivitis	0.477	0.022	1.61	1.07, 2.42

OR, odds ratio; 95%CI, 95% confidence interval.

This table, using multivariable logistic regression analysis and controlling for other variables, shows a significant positive association between OAB and allergic asthma, atopic dermatitis, urticaria, and allergic conjunctivitis.

## Discussion

Reports on OAB prevalence vary around the world. The global OAB prevalence in children aged 5–10 years is 5–12% (6). The prevalence of OAB in Japanese children in 2002 was 17.8% (13). In 2006, data from the Republic of Korea indicated that the overall prevalence of OAB in South Korean children between the ages of 5 and 13 was 16.59% (5). An epidemiological survey conducted in 2018 showed that OAB prevalence in children aged 5–14 years in China is 9.01% (7). In our study, the overall prevalence of children aged 5–14 years in Northeastern China is 10.7%, which is lower than that of Japan and the Republic of Korea, and higher than the epidemiological data of China in 2018. Moreover, the prevalence rate tends to decrease with age, with the peak occurring between the ages of 5 and 8 years, which is consistent with the results of Chung et al. (5). There is no significant difference in OAB prevalence between the sexes.

Many potential risk factors for OAB in children are unknown. Previous studies suggest that constipation (14) and hyperactivity disorder (15) may be risk factors for OAB, which was further confirmed in our study. Based on a large amount of clinical work experience and preliminary data, it has been found that allergic factors are closely related to the occurrence and development of OAB in children (10, 16). Soyer et al. studied 178 asthmatic children under the age of 6 and found that children with asthma had an increased risk of frequent micturition and urgent urination. Another study on the relationship between the severity of asthma and lower urinary tract symptoms in adult men showed that the symptoms of abnormal urination (including frequent micturition, urgent urination, and nocturia) and urinary retention (including tension, weakness, intermittence, and incomplete emptying) were significantly higher in the asthma group compared with the subjects in the non-asthma group (17, 18). However, as clinicians are usually confronted with sick children rather than healthy individuals, there is a risk of population bias. Therefore, we conducted this community population survey, and the same results were obtained. Our study indicates that allergic asthma, anaphylactic conjunctivitis, atopic dermatitis, and urticaria are all risk factors for OAB.

Researchers have observed in sensitized animal models that subjecting the bladder mucosa to substances known to sensitize the bladder can induce increased urination frequency, decreased urinary pressure, and decreased urinary volume. These findings align with the symptoms commonly observed in individuals with OAB (19, 20). Furthermore, our previous research has also confirmed that OAB can be effectively alleviated by avoiding allergens in combination with antihistamines (10–16). An increasing body of data suggests the involvement of allergic factors in the onset and progression of OAB.

It is generally accepted that the pathogenesis of OAB is multifactorial. The duration, clinical manifestations, and pathogenesis of OAB in children differ from those observed in adults. Previous research has suggested several pathophysiological mechanisms, such as detrusor instability, bladder sensitivity, urethral and pelvic floor muscle dysfunction, abnormal mental behavior, and hormone metabolism disorders. Recent research has revealed that allergic variables significantly contribute to the initiation and progression of OAB. Research has demonstrated the presence of mast cells in the urothelium, lamina propria, and smooth muscle layer of the bladder wall. Furthermore, there is an elevated number of mast cells in individuals with OAB (21). Mast cells have the ability to not only directly mediate allergic or inflammatory responses but also release MCT to regulate neuronal activity through PAR2. PAR2 belongs to the G-protein-coupled receptor family and has been shown to be expressed on bladder C-fibers. PAR2 promotes increased sensitivity of bladder afferent nerve fibers, such as bladder C-fibers, leading to bladder overactivity. Studies have additionally demonstrated that the activation of mast cells causes the release of histamine (22). Histamine can increase the baseline tension of both the lamina propria and the detrusor muscle within the bladder. Additionally, it can increase the sensitivity of afferent nerves to bladder stimuli through the activation of H1 receptors, thereby inducing bladder hypersensitivity and excessive contractions (23, 24).

Although not a fatal condition, OAB represents a serious public health burden that causes many social and psychological problems for the patients and negatively affects their quality of life. Although some cases of OAB may resolve on their own, the incidence of frequent micturition, urgent urination, and urinary incontinence in adults is significantly higher if they have a history of OAB as children (25). In addition, studies have shown that the cure rate for bladder dysfunction decreases after the age of 10–12 years. Therefore, patients who do not recover by this age gap may have an increased chance of developing adult Lower Urinary Tract Syndrome (LUTS) (26, 27). Therefore, it is essential to understand the pathogenesis and related risk factors of OAB. Finding the role of allergic factors in OAB occurrence may have clear theoretical and practical uses. The identification of allergy-related risk factors may provide new ideas for the prevention and treatment of OAB. Our results indicate that allergic diseases are a significant risk factor for OAB in children. Based on these findings, clinicians may identify children with a history of allergic diseases earlier as a high-risk group for OAB and monitor them more closely. Managing allergic diseases effectively through the avoidance of known allergens and the use of antihistamines to control OAB symptoms can significantly improve patients’ quality of life and reduce the social and psychological issues associated with urgency, frequency, and incontinence.

Our study has some limitations. First, the sample size was small, and since the study was based on a community survey, there may be selection bias; the participants may not fully represent the entire target population. Additionally, the study relied on questionnaires completed by parents or legal guardians, which may be subject to subjectivity, potentially affecting the accuracy and reliability of the data. Second, the study was conducted over a period of just 1 month, with no clinical follow-up, limiting our ability to gather more extensive data and observe long-term trends. Finally, no mucosal biopsies were performed on either the OAB or control group participants, which could have provided more substantial evidence of mast cell presence.

## Conclusion

OAB prevalence among children in Northeastern China is 10.7%. Allergic diseases, such as allergic asthma, anaphylactic conjunctivitis, urticaria, and atopic dermatitis may significantly increase the likelihood of developing OAB. The identification of allergy-related risk factors may provide new ideas for the prevention and treatment of OAB.

## Data Availability

The original contributions presented in the study are included in the article/supplementary material, further inquiries can be directed to the corresponding author.
